# Strain analysis for the identification of hypertensive cardiac end-organ damage in the emergency department

**DOI:** 10.1186/s13089-018-0110-7

**Published:** 2018-11-19

**Authors:** Pavitra Kotini-Shah, Susana Cuadros, Felix Huang, Joseph S. Colla

**Affiliations:** 1 Department of Emergency Medicine, University of Illinois at Chicago, 808 S. Wood Street (MC 724), Chicago, IL 60612 USA; 2 University of Illinois at Chicago, College of Medicine, Chicago, USA

## Abstract

Hypertensive emergency is a life-threatening state. End-organ damage affecting the heart accounts for up to 52% of hypertensive emergencies commonly encountered in the emergency department. Recent evidence indicates that strain echocardiography with computerized speckle-tracking is more sensitive at identifying hypertension induced changes in the left ventricle (LV) mechanical function than traditional 2-D echocardiography. We present a case demonstrating the use of emergency physician performed point-of-care strain echocardiography to identify and quantify LV mechanical dysfunction during a hypertensive crisis and to monitor improvement over 6 h.

## Background

An estimated 26% of ED patients will have an elevated BP with half of these patients having a BP > 160/100 [1, 2]. Distinguishing hypertensive urgency and emergency is challenging as both conditions involve marked elevation of blood pressure (BP, i.e., systolic BP ≥ 180 and/or diastolic BP ≥ 120). Hypertensive emergency is a life-threatening state with evidence of end-organ damage, as opposed to hypertensive urgency, and requires rapid reduction in BP [3, 4]. The heart is the most common organ affected, accounting for 52% of hypertensive emergencies [3]. Strain echocardiography is a relatively new way to measure left ventricular (LV) mechanical function. Unlike conventional 2D echocardiography which relies on visual estimation to assess cardiac contractility, strain echocardiography utilizes computerized speckle-tracking to measure actual tissue deformation of the myocardium. Strain can be measured in the longitudinal, radial and circumferential planes of the left ventricle (LV). Peak longitudinal strain (PLS) is a measurement of the overall longitudinal deformation of the left ventricle from diastole to systole. PLS can be measured from clips captured from standard apical views of the heart [5]. Longitudinal strain has been shown to be more sensitive at identifying hypertensive changes in LV mechanical function than ejection fraction (EF), fractional shortening, mitral E/A ratio, and tissue Doppler mitral annular velocity [5, 6]. Normal strain measurements range from − 15 to − 22% [7]. We present a case where emergency physician (EP) performed point-of-care PLS analysis is used to monitor improvement in LV mechanical function over time for a patient with hypertensive cardiac emergency.

## Case report

**Fig. 1:**
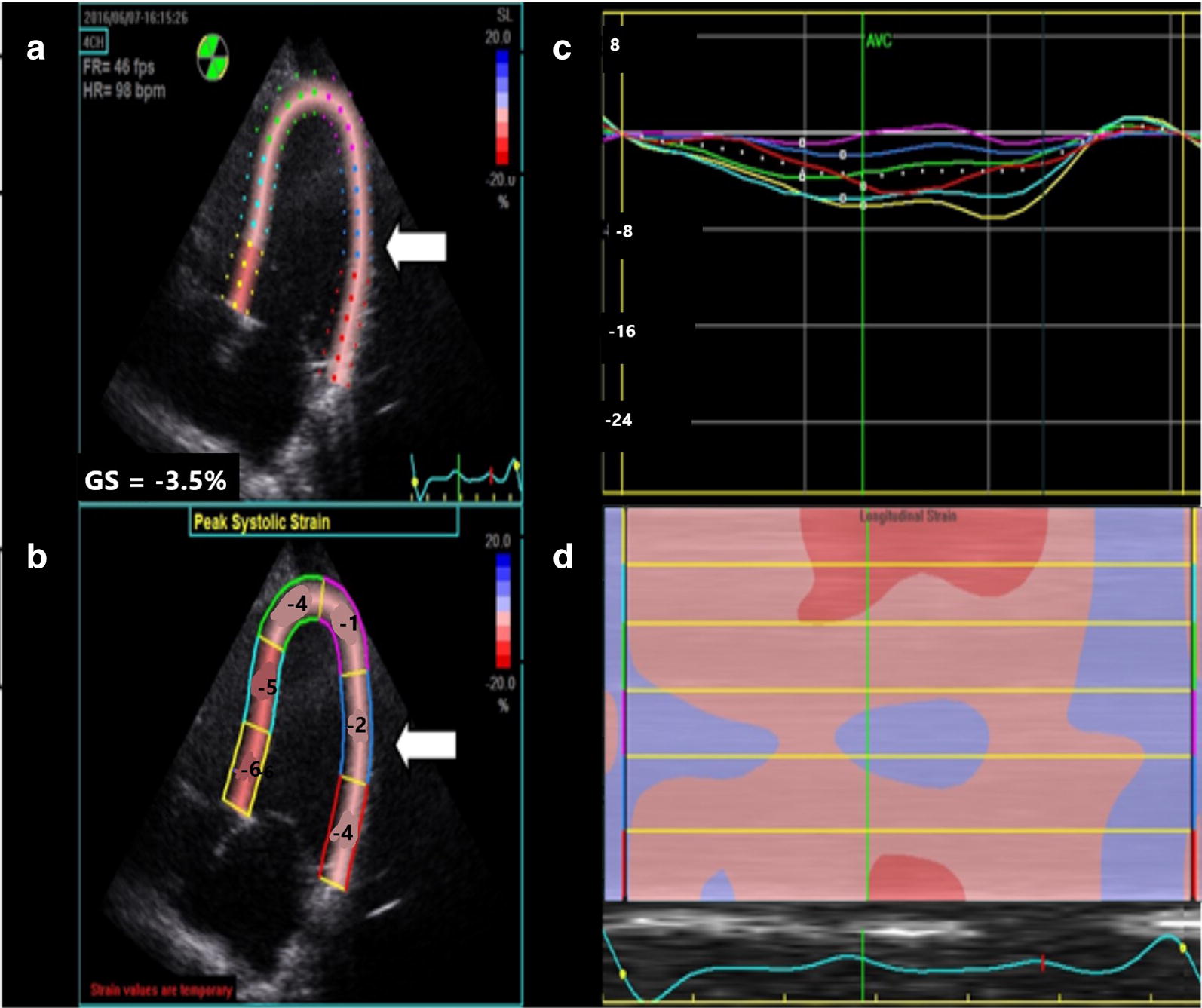
Quad display of peak longitudinal strain of patient before treatment

**Fig. 2:**
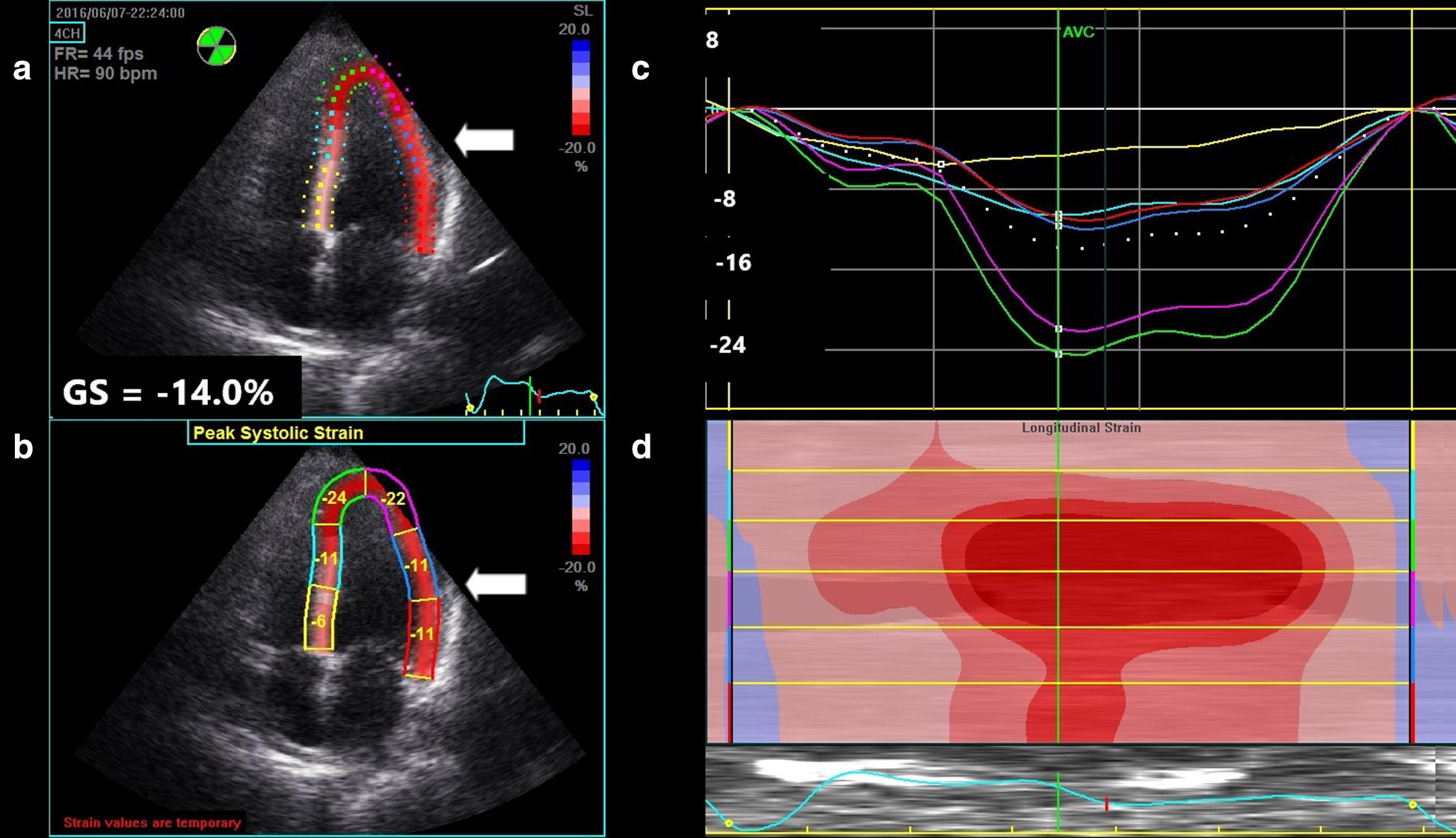
Quad display of peak longitudinal strain of patient after treatment

A 41-year-old woman with past medical history of peripartum cardiomyopathy, mitral regurgitation, and hypertension was referred to the emergency department (ED) due to severely elevated blood pressure. Patient reports a one week history of dyspnea, mild chest pressure with exertion, and stated that she had similar symptoms before with her pulmonary embolism more than 10 years ago. Three days prior to the onset of her symptoms, the patient had stopped taking her prescribed hydrochlorothiazide (HCTZ) due to increased urinary frequency, but maintained compliance with losartan. Vital signs at presentation were temperature 37.2 °C, BP 218/150, heart rate 121, respiratory rate 16, and pulse oximetry 100% on room air. On exam, the patient was well-appearing and in no apparent distress. The patient’s lungs were clear to auscultation and she had no S3, jugular vein distention, or lower extremity edema. The remainder of the physical exam was unremarkable. Further testing included labs, an electrocardiogram (EKG), a chest radiograph, and bedside echocardiogram (BSE) performed by ultrasound trained EPs. An apical four-chamber was obtained to calculate peak longitudinal strain (PLS) using only this view. Two initial troponin levels were mildly elevated at 0.08 µg/L, but down-trended thereafter to 0.05 µg/L. There was mild cardiomegaly and increased pulmonary vasculature on chest x-ray, and a new left bundle branch block (LBBB) on EKG. At the time of the initial BSE, BP was 252/163 [mean arterial pressure (MAP) = 170)] and PLS was − 3.5% (Fig. 1). The EF was not calculated, but estimated to be mildly reduced. Six hours later, the BP was 171/94 (MAP = 123) and a repeat BSE was performed and PLS was recalculated. Between the first and second BSE, the patient had received a total of 60 mg IV labetalol, 25 mg PO HCTZ, 40 mg IV furosemide, and was on a nitroglycerine drip at 40 mcg/min. The MAP had been reduced by 27% and the PLS improved to − 14% (Fig. 2). Repeat EKG after the IV medications continued to show a persistent LBBB. The patient was admitted to the cardiac intensive care unit for hypertensive emergency and acute coronary syndrome rule-out. Follow-up outpatient notes indicate that the patient was discharged home the following day and did not get re-admitted to the hospital within 30 days.

Figure 1 reflects the patient before hypertension treatment and Fig. 2 reflects the patient after treatment. Both Figs. 1 and 2 are quad displays of an apical 4-chamber image demonstrating peak longitudinal strain (PLS) of the left ventricle (white arrow). In each figure, top left image (A) is a 2D depiction showing the color coding for each left ventricle (LV) segment and the PLS in the 4-chamber view; bottom left image (B) displays the peak systolic strain for each of the six LV segments in the 4-chamber view; top right image (C) displays strain (*y*-axis) plotted over time (*x*-axis) for each of the six color-coded LV segments in a linear graphical display. The white dotted line shows the average of the six strain curves; bottom right image (D) shows the anatomical M-mode display depicting instantaneous strain for the 4-chamber plane with each LV segment color-coded on the *y*-axis, where the red color represents more negative strain.

## Discussion

Hypertensive emergency is a life-threatening disease with visits more than doubling from 2006 to 2013 [8]. Identification of cardiac end-organ damage requires high clinical suspicion and correlation with abnormalities in either EKG, cardiac markers or chest X-ray [9]. Two-dimensional transthoracic echocardiography is helpful, but requires more time to complete, and is not available to emergency departments at all hours. In addition, the delay from image acquisition by a technologist and image interpretation by a cardiologist limits its usefulness for monitoring cardiac function in the ED [10]. Once a good sonographic apical window is obtained at the bedside, an objective computer-generated strain percentage can be acquired by using speckle tracking software, eliminating the need for subjective visual estimation of EF. This advantage of strain analysis will likely decrease rates of LV EF misinterpretation. Furthermore, longitudinal strain provides information about both diastolic and systolic function and does not require complex Doppler measurements to estimate diastolic dysfunction [6, 11].

In the ED, it has been demonstrated that strain imaging can be used as a viable marker of response to treatment of acute heart failure, using ED re-admissions as a marker of adverse outcome [10, 12]. Strain analysis is also a viable method to monitor improvement of LV mechanical function after treatment of systemic hypertension [6, 11, 13, 14]. This case report supports recent papers suggesting potential role for strain analysis in evaluating heart disease in the ED setting.

Our patient demonstrated a three-fold improvement in LV strain, approaching a normal LV strain value, after antihypertensive and pre-load reducing treatment were given. Despite the improvement in PLS value observed within 6 h, the LBBB on EKG did not improve until the next morning. This suggests that the improvement in the electrical conductivity of the heart lagged behind the improvement of the mechanical deformation of the myocardium in this patient. Prior studies have suggested that strain imaging can detect alterations in the LV mechanics at the earliest phase of hypertensive disease even in asymptomatic patients [13]. However, this case is the first to document that subtle adaptive changes of the myocardium may precede changes depicted by conventional screening modalities such as EKG.

## Conclusion

Peak longitudinal strain analysis shows promise as an objective parameter for emergency physicians to monitor improvement of LV function during treatment for hypertensive cardiac emergency. This case report is the first to demonstrate a novel clinical application of this echocardiographic tool in the ED. Further study and documentation of PLS along the spectrum of hypertension is needed to better understand the myocardial dynamics at the patient’s bedside.

## Abbreviations

BP: blood pressure
BSE: bedside echocardiogram
EP: emergency physician
ED: emergency department
EF: ejection fraction
EKG: electrocardiogram
PLS: peak longitudinal strain
HCTZ: hydrochlorothiazide
LBBB: left bundle branch block
LV: left ventricle
MAP: mean arterial pressure
